# Delayed presentation of biopsy-proven eosinophilic myocarditis following COVID-19 mRNA vaccine

**DOI:** 10.21542/gcsp.2023.10

**Published:** 2023-05-11

**Authors:** Chaitra Janga, Tirth Patel, Hussam Al Hennawi, Sarin Atam, Shreeja Shah, Ifrah Naeem, Rahat Memon, Donald C. Haas

**Affiliations:** Department of Internal Medicine, Jefferson Abington Hospital, Abington, PA, USA

## Abstract

Myopericarditis associated with COVID-19 mRNA vaccines has been recognized as an uncommon adverse reaction, especially among young, healthy adult males. Eosinophilic myocarditis is a rare form of inflammation reflecting a hypersensitivity reaction following an inciting event commonly caused by drugs including vaccines. Eosinophilic myocarditis, a subtype of myocarditis, is characterized by eosinophilic myocardial infiltrates. It is usually accompanied by systemic eosinophilia in the form of a drug reaction with eosinophilia and systemic symptoms (DRESS) syndrome and is rarely associated with myocyte fibrosis and/or necrosis. In this report, we present a case of biopsy-proven eosinophilic myocarditis in a 24-year-old male patient, likely secondary to COVID-19 mRNA vaccination. To our knowledge, this is the first report to describe delayed eosinophilic myocarditis following the COVID-19 mRNA vaccine. Clinicians should be aware of possible delayed presentation to avoid associated morbidity.

## Introduction

Eosinophilic myocarditis is a rare inflammatory cardiomyopathy associated with other hypereosinophilic syndromes, hypersensitivity myocarditis, eosinophilic granulomatosis with polyangiitis, infections, malignancy, toxins, and idiopathic myocarditis. Driven by hypersensitivity reactions, hypersensitivity myocarditis can be caused by a wide array of inciting events. These may include antibiotics (36.5%), central nervous system agents (21.1%), vaccines including non-mRNA (7.7%), antitubercular drugs (1.9%), and a variety of other drugs (32.8%)^1^.

Of note, no clear histological pattern is associated with COVID-19 mRNA vaccination-induced myocarditis; nevertheless, eosinophilic myocarditis is a histological diagnosis and requires an endomyocardial biopsy. However, there are only a few case reports of biopsy proved COVID-19-mRNA-vaccination induced EM to date. Nevertheless, there is increasing evidence that COVID-19 mRNA vaccine-induced myocarditis is a hypersensitivity myocarditis with or without systemic eosinophilia^2–14^. To our knowledge, myocarditis following COVID-19 mRNA vaccination, with subsequent biopsy-proven eosinophilic myocarditis, has been reported in at least 12 patients^15–23^. Most reported cases describe COVID-19 mRNA vaccine-induced myocarditis occurring in the first 2 weeks following COVID-19 mRNA vaccination^15–23^. Our case highlights the first case of delayed hypersensitivity reaction consistent with EM 3 months after the second dose of the COVID-19 mRNA vaccine.

## Case presentation

A healthy 24-year-old male with history of allergic rhinitis presented with chest tightness, dyspnea, and hemoptysis. He denied recent infections, travel, changes in diet, medications, or alcohol consumption and had two cats at home. He was exposed to his family with COVID-19 infection six months prior to presentation; however, he was asymptomatic and never tested positive. Notably, he received a second dose of the Moderna vaccine (mRNA-1273) three months before presentation.

**Figure 1.**
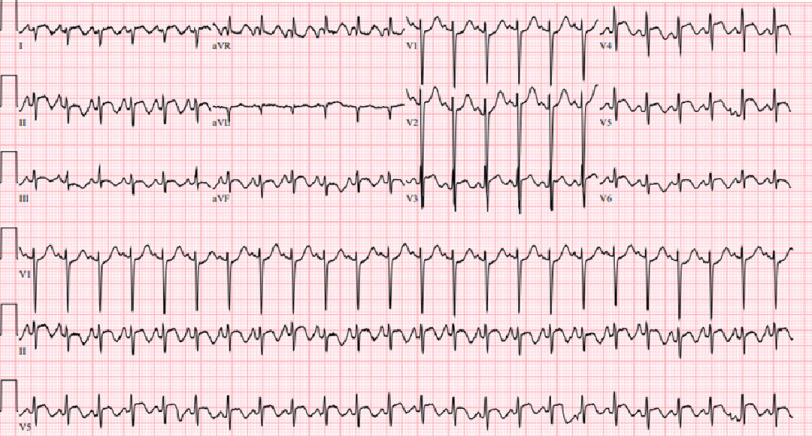
ECG with diffuse ST-segment elevation and T-wave inversion.

On arrival, the patient was hypoxic to 88% on room air, with a heart rate of 133 bpm, temperature 101.5 F, and blood pressure of 122/68 mmHg. On examination, he was in acute distress, diaphoretic, and tachycardic with a regular rhythm. Pulmonary auscultation revealed diffuse bilateral rhonchi, with the remainder of the examination being negative for rashes and lymphadenopathy. Electrocardiography (ECG) showed sinus tachycardia with diffuse ST-segment elevation and T-wave inversion (Figure 1).

Chest radiography revealed a clear lung. Initial troponins were 6629 ng/L (<19 ng/L), pro-BNP 5577 pg/ml ( ≤ 125 pg/ml), and creatine kinase (CK-MB) 1038 IU/L (25–125 IU/L). Other lab work included WBC 16.4 B/L (3.4–10.8x10E3/uL), with 70.6% neutrophils and 0.60 absolute eosinophils, BUN 15 mg/dL (6–20 mg/dL), creatinine 1.23 mg/dL (0.26–1.27 mg/dL), ALP 65 IU/L (29–92 IU/L), AST 137 IU/L (7–42 IU/L), ALT 47 IU/L (<45 IU/L), lactate 1.6 mmol/L.

Inflammatory markers were elevated at ESR 55 mm/h and CRP 8.70 mg/dL. Bedside echocardiography revealed a decreased ejection fraction of approximately 35–40%. In retrospect, he had an abnormal ECG consistent with sinus tachycardia with fusion complexes and T-wave inversions in the inferior and lateral leads two weeks before presentation, during regular physical examination for job application. During that visit, the echocardiogram showed normal LV function with EF 55–60%, and the patient was asymptomatic until the current presentation. He had normal ECGs approximately two years ago, without any known underlying heart disease.

**Figure 2.**
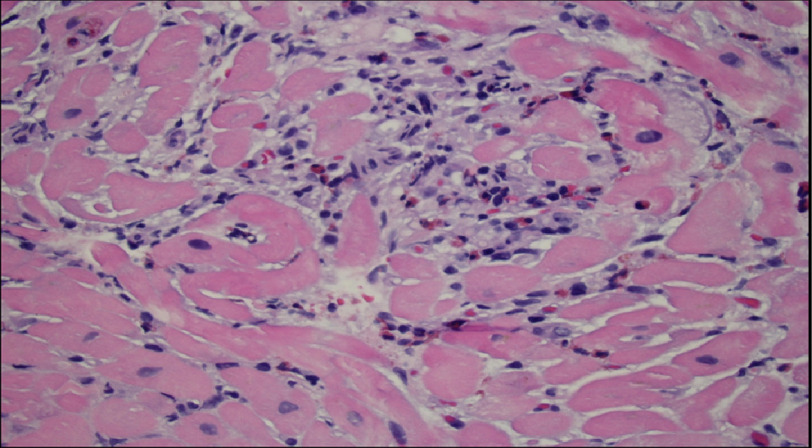
Myocardial biopsy showing a focal inflammatory area composed predominantly of eosinophils (cells with red granular cytoplasm), admixed with lymphocytes and rare neutrophils.

One day after the initiation of IV methylprednisone, a repeat echocardiogram showed a preserved EF of 45–50% with grade 3 diastolic dysfunction. Right heart catheterization was unremarkable, with normal filling pressures and cardiac index, excluding pulmonary embolism. An endomyocardial biopsy was performed, which showed myocarditis with prominent eosinophils (Figure 2). In the interim, due to normal heart filling pressures, suspicion of diffuse alveolar hemorrhage was high, with hemoptysis on presentation. Bronchoscopy ruled this out, and bronchioloalveolar fluid studies were negative.

An extensive workup was performed: infection (HIV, EBV, CMV, COVID, Coxsackie, Giardia, cryptosporidium, aspergillus, histoplasma, strongyloides, trichinella, quantiferon), autoimmune causes (IgE, ANA, ANCA), and hypersensitivity pneumonitis were all ruled out (Table 1). The likely etiology of eosinophilic myocarditis in our patient was a delayed hypersensitivity reaction following the COVID-19 mRNA vaccine. The patient was treated with a pulse dose of methylprednisone (1 g intravenously for three days.

**Table 1:** Laboratory tests for myocarditis workup.

Laboratory test	Result
Lyme disease antibody	Negative
Syphilis antibody, Treponemal pallidum antibody cascade screening test	Negative
Mycobacterium TB gold	Negative
Chlamydia trachomatis TMA, rectal	Negative
HSV 1, 2 DNa	Negative
Parvovirus B19 IgM, IgG	Negative
Influenza A, B	Negative
HIV Ab/Ag	Negative
Hepatitis panel	Negative
SARs CoV-2 RT PCR	Negative
SARS-CoV-2 anti nucleocapsid antibody	Positive
SARS-CoV-2 anti spike glycoprotein antibody	Positive
Coxsackie B 1-6 antibody	Negative
Enterovirus RNA, real-time PCR	Negative
Respiratory pathogen panel	Negative
Varicella zoster IgG	Negative
Giardia Cryptosporidium TIA	Negative
Toxoplasma IgM/ IgG antibody	Negative
Cryptosporidium antigen	Negative
Histoplasma antigen candidate	Negative
trichinella antibody IgG	Negative
Strongyloides antibody IgG	Negative
Thermoactinomyces vulgaris IgG	Negative
Hypersensitivity pneumonitis screen	Negative
Asperigillus galactomannan antigen	Negative
Giardia lamblia	Negative
Neisseria gonorrhea TMA, rectal	Negative
Streptococcus pneumoniae antigen	Negative
Asperigillus fumigatus	Negative

A repeat echocardiogram on the third day of steroid treatment showed normalization of EF to 60–65%, with an improvement in ECG findings. He was discharged on a steroid taper, 50 mg oral methylprednisone with a decrease in 10 mg dose every seven days for his eosinophilic myocarditis, along with lisinopril and carvedilol. At a follow-up visit a month later, the patient reported improvement in chest tightness and dyspnea. However, he also reported intermittent palpitations. His mobile outpatient telemetry showed a high burden of premature ventricular contraction (PVC) with transient ST-segment and T-wave abnormalities; hence, a loop recorder was placed for prolonged cardiac monitoring.

## Discussion

We present a novel case of biopsy-proven EM following COVID-19 mRNA (Moderna) vaccination in a young, healthy male without systemic eosinophilia, highlighting the possibility of delayed hypersensitivity myocarditis following vaccination. Myocarditis is a diverse disease with varying clinical presentations attributed to different etiologies and therapeutic effects caused by an inflammatory reaction to the myocardium without ischemia. Viral infections, including SARS-CoV-2, are the most common triggers of myocarditis, whereas other etiologies may include drug reactions and vaccine exposure^24,25^.

Eosinophilic myocarditis (EM) is a rare type of inflammatory cardiomyopathy, characterized by eosinophilic infiltration of the myocardium. The incidence of EM is unclear thanks to non-specific diagnostic criteria and a reliance on endomyocardial biopsy for diagnosis. However, the mortality rate seems to be high, at approximately 20%^26^. Therefore, early recognition and prompt management of EM is vital for reducing the disease burden. Approximately one-third of EM cases are caused by hypersensitivity reactions, with the likely etiology being vaccines and medications^26,27^. There have been some reported cases of post-vaccination eosinophilic myocarditis after smallpox, diphtheria, tetanus, and pertussis vaccination^28,29^. Such non-mRNA vaccine-induced EM can also be observed following conjugate meningococcal C and hepatitis B vaccines in children^15^. Recently, reports of EM secondary to COVID-19 mRNA vaccination have been documented, with few fulminant cases^15–23^.

To date, myocarditis following a COVID-19 mRNA vaccine has been described in several cases. Among the few patients who underwent endomyocardial biopsy, approximately 11 cases of EM have been reported. The cases were reported following a Pfizer vaccine or for five cases, including our patient, after the Moderna vaccine. Among the reported cases following the Moderna vaccine, all were diagnosed within a week or two of receiving the COVID-19 mRNA vaccine^15–23^. In contrast, our patient received the vaccine approximately three months before the clinical presentation, indicating a possible delayed hypersensitivity reaction to the COVID-19 mRNA vaccine.

The clinical presentation of EM varies from the typical symptoms of chest pain, dyspnea, syncope, palpitations, and fever to non-specific symptoms, and the spectrum varies from mild asymptomatic disease to cardiogenic shock^30,31^. Historically, endomyocardial biopsy (EMB) has been the gold standard for diagnosing EM with a sensitivity of approximately 50%^32^. However, the utilization of EMB has decreased due to the widespread utility of MRI in diagnosing myocarditis and is used only in exceptional cases of rapid clinical deterioration, as in our patient. EM diagnosis depends on the histological pattern of inflammation, which can be revealed only by performing EMB^33^. In our case, myocarditis was suspected based on the clinical presentation supported by the elevated troponin, CK-MB, and echocardiography findings. However, due to rapid deterioration of the patient, coronary angiography with EMB was performed, which revealed EM. Our patient did not have any exposure to drugs or other vaccines, no evidence of infection, or autoimmune condition, supporting the etiology of the delayed hypersensitivity reaction from the COVID-19 vaccine. The treatment of EM secondary to COVID-19 mRNA vaccines remains unclear. Literature has shown steroids to be useful in cases of COVID-19 mRNA vaccine-induced myocarditis, as it is believed to be secondary to hypersensitivity reaction^19^.

## Conclusion

Early onset hypersensitivity reactions in the form of myocarditis have been reported after administration of the COVID-19 mRNA vaccine. This case highlights the first delayed hypersensitivity reaction in the form of EM in a healthy patient months after administration of the CVOID-19 mRNA vaccine. Clinicians should be aware of the delayed nature of this association, as it may mimic other severe cardiac diseases. Appropriate evaluation and timely intervention are essential in cases of high suspicion for COVID-19 mRNA vaccine-associated myocarditis, as an aggressive strategy warrants survival, as in our case. Despite the reported adverse effects of COVID-19 vaccination, its benefits still outweigh the risks; therefore, vaccination is still recommended with close monitoring.
